# Transitions Between Compensated Work Disability, Joblessness, and Self-Sufficiency: A Cohort Study 1997–2010 of Those Jobless in 1995

**DOI:** 10.1007/s11113-016-9412-2

**Published:** 2016-09-14

**Authors:** Michael Wiberg, Staffan Marklund, Kristina Alexanderson

**Affiliations:** 1 Division of Insurance Medicine, Department of Clinical Neuroscience, Karolinska Institutet, 171 77 Stockholm, Sweden; 2 Department of Analysis and Forecast, Swedish Social Insurance Agency, Stockholm, Sweden

**Keywords:** Work, Unemployment, Sick leave, Disability pension, Multi-state, Cohort study

## Abstract

Associations between unemployment, work, and disability have been researched in many studies. The findings are often based on cross-sectional data and single outcomes. The present study analysed multiple outcomes over a period of 15 years among long-term unemployed individuals. Based on all individuals aged 20–40 living in Sweden in 1995, prospective cohort analyses were conducted. Individual annual labour market proximity 1995–2010 was estimated and categorised into three mutually exclusive categories: “Jobless”, “Self-sufficient” (i.e. main income from work), or “Disabled”. Individuals in the category “Jobless” (*n* = 638,622) in 1995 constituted the study population. Using autoregressive multinomial logistic regression, transitions between the three states during 1997–2010 were analysed. Socio-economic factors, previous inpatient care, and national unemployment rates in different time periods were included in the regression models. Among those “Jobless” in 1995, 17 % were also “Jobless” in 2010, while 10 % were “Disabled” and 61 % “Self-sufficient”. The transitions were stable over time periods for transitions into “Self-sufficient” and “Disabled” but less so for “Jobless”. Previous state was the best predictor of subsequent state. “Jobless” individuals with previous morbidity had a higher transition probability into “Disabled” and a lower transition probability into “Self-sufficient”. The transition rates into “Self-sufficient” were higher in periods with lower unemployment levels. The study supports the interpretation that return to work was affected both by the individuals’ previous health status and by the national unemployment level. Transition from being “Jobless” into “Disability” may be influenced by previous ill health and by negative health effects of being “Jobless”.

## Background

Several studies have shown associations between aggregated unemployment rates and sickness absence rates in different countries (Allebeck and Mastekaasa 2004; Henrekson and Persson 2004; Lidwall and Marklund 2011; SIA 2014). Generally, sickness absence rates have increased when unemployment rates decreased and vice versa (Allebeck and Mastekaasa 2004; Lidwall and Marklund 2011; SIA 2014). Holland and collaborators studied macro-level contexts in five countries and found that general de-industrialisation trends affected employment prospects of chronically ill and disabled, but that recession did not effect this (Holland et al. 2011a). This mechanism was partly reduced in countries with greater investments in active labour market policies (Holland et al. 2011b).

Similarly, numerous studies based on micro-data have indicated negative associations also between individual unemployment experience and risk of sickness absence or disability pension (Allebeck and Mastekaasa 2004; Johansson and Palme 1996; Lidwall et al. 2004). Longer time in unemployment has been shown to affect the risks not only of future unemployment but also of sickness absence and disability pension (Allebeck and Mastekaasa 2004; Andrén 2008; Bäckman and Franzén 2007; Hanisch 1999; Helgesson et al. 2012; Janlert and Hammarström 2009; Johansson and Palme 1996; Kokko et al. 2000; Lidwall et al. 2004; McKee-Ryan et al. 2005; Milner et al. 2014; Montgomery et al. 1996; Schmitz 2011).

Most research on the associations between unemployment and sickness absence has been based only on the factors employment, unemployment, or health-related work incapacity, while other possible labour market states have not been taken into consideration. Old-age pension, educational, paid parental leave, and income support in the form of a poverty allowance are examples of such states. If a wide range of possible outcomes are not simultaneously included in the analyses, there is a risk that the complexity of the movements between labour market positions will not be fully recognised.

However, a few studies have jointly analysed several labour market outcomes. Some have analysed multiple transitions of individuals from sickness absence to work, unemployment, and disability pension (Lie et al. 2008; Øyeflaten et al. 2012, 2014; Pedersen et al. 2012, 2014). Lie and collaborators studied sickness absentees who had been subject to an intervention with the three outcomes: continued sickness absence, return to work, and disability pension (Lie et al. 2008). Pedersen and collaborators studied transitions between sickness absence, work, unemployment, and disability pension of individuals, who at inclusion were sickness absent (Pedersen et al. 2012). In a similar study, Pedersen et al. showed that the introduction of socio-economic confounders only moderately improved the accuracy in predicting future states of the sickness absentee (Pedersen et al. 2014). However, a study by Oyeflaten and collaborators on return to work among sickness absentees after rehabilitation found that a range of socio-economic and health indicators affected the transitions between work, sickness absence, and disability pension (Øyeflaten et al. 2014). Similarly, Steiner found that individual heterogeneity strongly affected the transition rate into reemployment and that economic recession affected these differences (Steiner 2001). A study by Alba-Ramirez and collaborators indicated that duration of unemployment affected the transition rate of finding a new employer but not the rate of returning to the same employer (Alba-Ramírez et al. 2007).

The refereed studies have additionally shown that the initial state is of prime importance for the prognosis of future states. Furthermore, theory and macro-studies indicate that variations in macro-economic conditions also should be considered in this context. In macro-economic terms, a national low unemployment rate suggests that jobs are relatively easy to get and that the risks of being dismissed, e.g. because of absence may be lower. On the other hand, for any given macro-economic state there may be individual characteristics that contribute to determine the individual job outcomes.

Several interpretations of the association between work, unemployment, and health-related work incapacity have been proposed. For example, it has been suggested that people with ill health are at greater risk of losing their jobs during a recession, but return to work in times of economic prosperity (Allebeck and Mastekaasa 2003; Hesselius 2007; Leigh 1985; Virtanen et al. 2003). Another interpretation is that individual unemployment per se causes health problems that may lead to future sickness absence or disability pension (Janlert and Hammarström 2009). Logically, then, persons who might be able to return to work from sickness absence during periods of low unemployment may find low or no demand for their work capacity when the unemployment rate is high, i.e. when the demand for labour is low. This can increase the risk of transition to a disability pension. Thus, in order to get a picture of the influence of both health and aggregate unemployment levels on transitions between labour market states, information about previous health as well as information about variations in macro-economic conditions is essential to the analysis.

Handling transitions between multiple labour market positions over a long period of time is statistically challenging and the transition estimates will too soon be too many to comprehend. Thus, the number of possibilities needs to be simplified in line with a specific study aim.

A possible strategy is to reduce the number of labour market positions by adding similar positions to one another. For example, being unemployed, living on means-tested social assistance, and having no or minimal income from work may be amalgamated by assuming that they are all indications of joblessness or near joblessness of the individuals, but that they are considered to be available for work on the labour market. Similarly, being long-term sickness absent or disability pensioned may be merged since they are both based on health-related incapacity to work. Further, it is well known that disability pension is usually preceded by long-term sickness absence (Dorner et al. 2015; Karlsson et al. 2008; Kivimäki et al. 2007; OECD 2010).

In the present study, simplification of the complexity of individuals labour market positioning was operationalised by categorising individuals regarding their estimated distance to the labour market: (1) low/no income but available for work, (2) having the main income from work, and (3) not available for work due to disease/injury. These states were named “Jobless”, “Self-sufficient”, and “Disabled” (described in more detail below).

### Aims and Research Questions

The aim of this study was to examine individual transitions between self-sufficiency, joblessness, and work disability over a 15-year period in the Swedish population among individuals who were jobless at baseline. A further aim was to assess the degree to which these transitions were affected by individual factors or by macro-level conditions. Three research questions were examined:

1. How did individuals, classified as “Jobless” in 1995, move between “self-sufficiency”, “Joblessness”, “Disabled”, or leave the study population in the years 1997–2010?
2. How were these transitions between states associated with the individuals’ socio-demographic factors and previous health status?
3. How were transition probabilities associated with national labour market conditions?

## Materials and Methods

A prospective cohort study, based on multi-state analyses, was conducted.

**Fig. 1:**
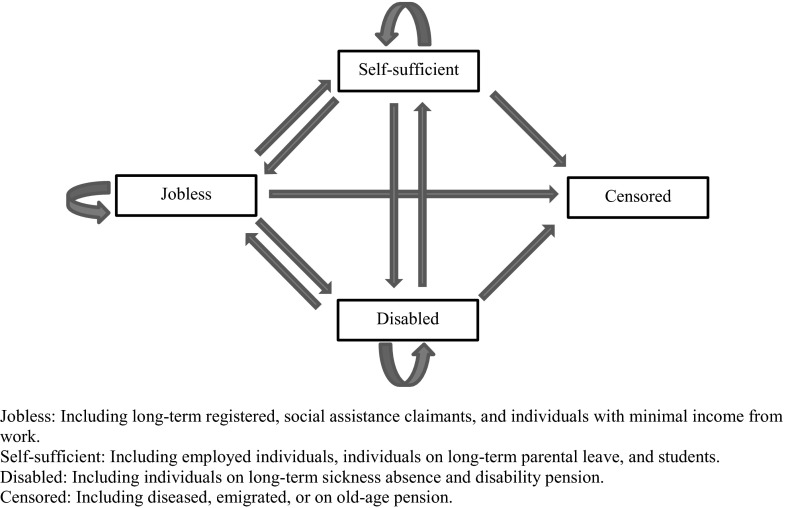
Model for transitions between the four states jobless, self-sufficient, disabled, or censored (absorbing state) 1997, 2001, 2005, 2008, and 2010. Entry: all jobless in 1995

As shown in Fig. 1, the study has focussed on individuals moving between three major labour market states and a censor category over 15 years. A prospective cohort study was conducted, based on data for all individuals 20–40 years of age and living in Sweden at the end of 1994 and 1995 (*n* = 2,514,464, Table 1). Frequencies and proportions are presented in Table 1. About 26 % (*n* = 638,622) were classified as “Jobless” in one of the three categories. The corresponding proportions for “Self-sufficient” and “Disabled” were 72 and 3 %.

**Table 1:** Main labour market positions among the women and men living in Sweden 1995, 20–40 years of age

	Women		Men		All	
	*n*	(%)	*n*	(%)	*n*	(%)
Jobless						
Registered long-term unemployed^a^	173,820	14	218,215	17	392,035	16
Social assistance^b^	19,473	2	22,568	2	42,041	2
Minimal income^c^	109,552	9	94,994	7	204,546	8
Self-sufficient						
Employed^d^	687,157	56	842,512	66	1,529,669	61
Long-term parental leave^a^	113,885	9	3098	>0	116,983	5
Students^b^	79,840	7	67,911	5	147,751	6
Disabled						
Disability pension^a^	22,913	2	21,516	2	44,429	2
Long-term sickness absence^a^	21,040	2	14,942	1	35,982	1
Retired						
Old-age pension^b^	544	>0	479	>0	1023	>0
All	1,228,224	100	1,286,235	100	2,514,459	100

^a^More than 6 months

^b^More than half of the total annual income

^c^No or minimal income from work or social security

^d^Categorised as employed/self-employed and having earnings above a fixed level, set by Statistic Sweden

The individuals who were in the state “Jobless” in 1995 constituted our study population (*n* = 638,622). They were followed annually during 1996–2010 by use of data from the nationwide register Longitudinal database for income and labour market studies (LISA) compiled by Statistics Sweden (SCB 2011) regarding annual income from work (i.e. earnings) and social security systems. Information on the individuals’ sex, age, educational level, country of birth, and year of emigration was also obtained from LISA. Additionally, annual number of inpatient days (that is, days spent in hospital inpatient care) and year of death were obtained for each individual from the National Board of Health and Welfare. Linkage of the different registers was performed by use of the unique personal identifier that is assigned to all Swedish residents.

### Classification of Labour Market Positions

Each individual in the study population was categorised into mutually exclusive labour market positions for each year 1995–2010. The classification was performed in two steps. In the first step, individuals were classified into nine different labour market positions (Wikman et al. 2012). This categorisation was based on definitions used by Statistics Sweden who classified individuals as working (including self-employment) or not, depending on the size of their annual earnings. A person whose earnings exceeded a minimal amount, annually calculated by Statistic Sweden, was categorised as working. The categorisation based on compensation from different social insurance programmes (parental leave, registered unemployment, sickness absence, and disability pension) was based on number of days of compensation. If the annual sum of compensated days for all the various benefits was at least six months, the individual was categorised to the relevant corresponding group. There was, however, no information about the number of days during the year that benefits were received for means-tested social assistance, student allowances, or old-age pensions. Therefore, those with more than half of their annual disposable income from either of these sources were categorised as social assistance recipient, student, or old-age pensioner, respectively. Further, categories for those who emigrated or died were defined for the follow-up years 1996–2010. Emigration was defined as not being a Swedish resident at the end of the studied year. Table 1 presents the numbers of women and men in the nine categories in 1995 in this first step of the categorisation.

In the second step, these categories were combined into the four mutually exclusive analytical categories mentioned in the introduction. These larger categories are referred to as “states” and were used for analysing transitions.

The first state was named “Jobless” and included registered long-term unemployed (i.e. ≥6 months) with unemployment compensation, those on social assistance, and others with no/low earnings or transfer from social security during a year. The second state was named “Self-sufficient”. It consisted of those with earnings predominantly from employment or self-employment during a year, but also individuals on long-term, compensated parental leave in conjunction with a newly born child, and individuals on student allowance. The third state, named “Disabled” was composed by individuals on long-term sick leave or on disability pension (≥6  months). Long-term sick leave and disability pension were both medically certified according to the Swedish social security legislation. The fourth state was referred to as “Censored”. This included those who died, emigrated, or became old-age pensioners. If an individual re-immigrated to Sweden he or she was not reintroduced into the study population.

### Follow-Up Periods

**Fig. 2:**
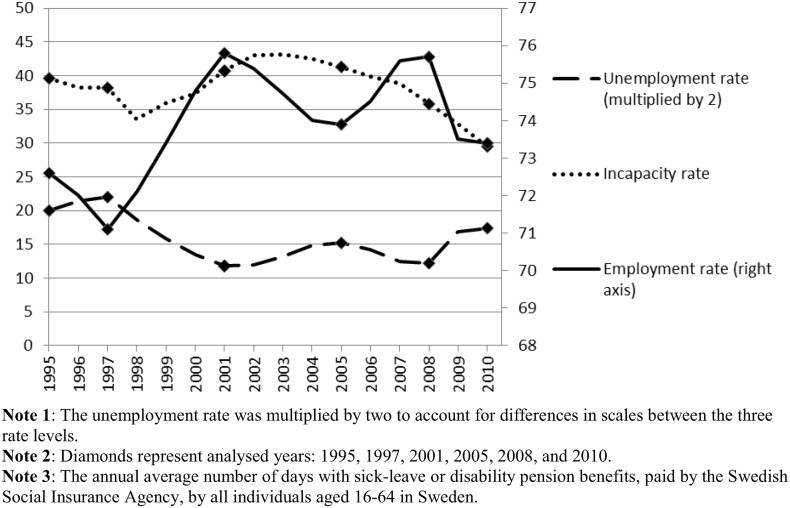
Unemployment rate, incapacity rate, and employment rate in Sweden 1995–2010. *Sources* The Swedish Social Insurance Agency and www.ekonomifakta.se

Categorising the population into mutually exclusive groups enabled us to follow changes in individuals’ labour market position from one year to another. The labour market states for the individuals who were “Jobless” in 1995 were followed up at the five specific years of 1997, 2001, 2005, 2008, and 2010. The main reason to study transitions in these years rather than to study all years was to be able to capture different macro-level conditions, according to the aim of the study. The five selected follow-up years mark years with increase or decrease of unemployment rates in Sweden for individuals aged 16–64. The rationale behind selecting these specific years was that transitions into long-term unemployment as well as into long-term sickness absence and disability pension may be affected by the availability of jobs and, specifically, the availability of jobs for individuals with reduced work capacity. Figure 2 shows macro-level data of the development of the three states, denoted by the unemployment rate, the incapacity rate (sick leave and disability pension), and the employment rate for the entire period examined, 1995–2010 (EkonomiFakta 2015a, b; SIA 2011).

## Statistical Analysis

Initially, transitions between the different states in 1997, 2001, 2005, 2008, and 2010 were computed as unadjusted frequencies. Thereafter, based on a multi-state framework, analyses were conducted to estimate the transition probabilities, including the effects of socio-economic factors and individuals’ prior health problems in terms of inpatient care, taking into account macro-economic unemployment levels.

### Multi-state Framework

Analyses, which allowed for several changes between states, were conducted for transitions between the states “Jobless”, “Self-sufficient”, “Disabled”, or “Censored” during the follow-up periods among the cohort of jobless individuals in 1995. The state “Censored” was defined as an absorbing state, meaning that no transitions were possible from that state. An illustration of the twelve distinct transitions between the four states at different points in time is presented in Fig. 1.

All models were designed as a first-order discrete time, discrete state Markov chain. A type of method which is centred around the idea that only information in specific parts of the temporal chain of events is relevant for describing subsequent events in the chain. In a first-order Markov chain, information of an object positioning at a certain time point is assumed to incorporate all relevant information for describing the objects subsequent position. Thus, adding information on the object’s previous positioning does not add new information. In the case of this study, this means that an individual’s following state is assumed to be independent of all previous states—given the information on the individual’s current state (de Vries et al. 1998).

Three models were estimated. In model 1, only the preceding state was included as an explanatory variable (reported for comparison with model 2 and 3). In model 2 and 3, covariates were also included. Thereby, model 1 was a pure first-order Markov chain with homogenous transitions probabilities (i.e. independent of time of measurement), whereas the two other models were based on less strict assumptions on the level of information in the state p-1 by including other variables that could affect the transition probabilities (Commenges 1999). The models were specified using two time scales: one for the measured period and one for actual annual time (1995, 1997, 2001, 2005, 2008, and 2010). The periods (*p*) corresponded to specific annual time points (*t*) accordingly: *p*_0_ = 1995, *p*_1_ = 1997, *p*_2_ = 2001, *p*_3_ = 2005, *p*_4_ = 2008, and *p*_5_ = 2010. The years 1997, 2005, and 2010 were characterised by peaks of national unemployment. The opposite was observed for the years 1995, 2001, and 2008.

A simplified theoretical representation of the models is presented below:

$${{\text{Model 1}:\,State_{p,i} = \alpha + State_{p - 1,i}}}$$

$${{\text{Model 2}:\,+ Sex_{i} + Age_{i} + Country_{i} + Education_{p - 1,i} }}$$

$${{\text{Model 3}:\, + Period + Inpatient care_{t - 1:t - 2,i}}},$$

where *State*_*p,i*_ and *State*_*p*−1,*i*_ represent individual’s i:s state (self-sufficient, jobless, disabled, or censored) in period *p* (*p*_1_ to *p*_5_), respectively, previous state (*p*_0_ to *p*_4_). The state-specific intercept is represented by *α* and was interpreted as the transition probability for individuals with sets of covariates defined at the reference category. Sex, Age (5-year intervals), and Birth country (Sweden/Other) were all measured in 1995, whereas level of education (Lower, High school, Higher) was measured at the time of the previous state. Further, “Inpatient care” represents the number of days of inpatient hospital care (with exception for normal child birth (ICD 10: O80), conditions for new-born/foetus originating in the perinatal period (ICD 10: P00-96), and persons encountering health care (mainly observations) (ICD 10: Z00-99) (WHO 1992) in the 2 years prior to the outcome. Inpatient care was categorised based on the median for those with any such care in the time period (0, ≤median, >median). It should be noted that inpatient care in general implies more serious conditions than outpatient or primary healthcare. Finally, dummy variables for the periods, which by design incorporated high/low levels of national unemployment rats, were described by “Period”.

### Empirical Models

Markov-chain models of the kind used here can be estimated through logistic regressions by using the preceding state to estimate the current state (thereby, an autoregressive logistic regression) (de Vries et al. 1998). Models 1–3 were operationalised using multinomial logistic regression, using “Jobless” as the reference category for both the present and prior state. According to the Markov assumption of conditional independence, repeated observations within an individual was considered independent. Further, since the state “Censored” was defined as an absorbing state, the models were estimated with the restriction that the previous state was not “Censored”. In each model, four outcomes were possible, meaning that the multi-nominal regression models jointly estimated three equations. This ensured that the sum of the estimated probabilities for the four different states was 100 %. Log-likelihood tests were used to compare model fit between the three models.

Further, based on the estimated parameters from Model 3, transition probabilities were calculated for specific sets of socio-demographic factors, and in low/high national level of unemployment, and in different individual levels of previous inpatient care, using the definitions described above.

## Results

### Description of the Study Population

At the start of follow-up in 1995, the studied cohort consisted of the 638,622 residents in Sweden in ages 20–40 classified as “Jobless” (53 % males) (Table 2). Their educational level was relatively low—16 % had attained a higher level of education (university level), compared to 26 % in the general population. The proportion of individuals born outside of Sweden (25 %) were higher than in the general population (13 %, figures not shown in tables). The majority, 86 %, did not have any inpatient care in the 2 years prior to 1995. At the end of the study 12 % had been censored (emigration, old-age pension, or death). The most notable change in socio-demographics over time was that gradually a higher proportion of the cohort had attained higher levels of education (31 % with higher education in 2010, compared to 16 % in 1995).

**Table 2:** Socio-demographics, previous inpatient care, and labour market state at inclusion (1995) and at the end of follow-up (2010), for the individuals who were jobless in 1995^a^

	1995		2010	
	*n*	(%)	*n*	(%)
Sex				
Male	335,777	52.6	292,668	52.1
Female	302,845	47.4	269,542	47.9
Age (mean)	29.5		43.4	
Education				
Low	159,250	24.9	78,993	14.1
High school	375,703	58.8	309,037	55.0
Higher	103,669	16.2	174,180	31.0
Born in Sweden				
Yes	477,083	74.7	437,061	77.7
No	161,539	25.3	125,149	22.3
Inpatient care, no. of days^b^				
0	551,465	86.4	493,893	87.9
≤Median^c^	46,957	7.4	38,756	6.9
>Median^c^	40,200	6.3	29,561	5.3
*N*	638,622		562,210	
State				
Jobless	638,622	100	109,972	17.2
Self-sufficient	0	0	387,771	60.7
Disabled	0	0	64,467	10.1
Censored	0	0	76,412	12.0

^a^Including registered long-term unemployed, individuals on social assistance, and individuals with a minimal work income. For 2010, old-age pensioners, deceased, or emigrates after 1995 were excluded

^b^Inpatient hospital care; 1993–1994 for 1995, and 2008–2009 for 2010

^c^Calculation based on those with such care

Table 3 describes the proportion of transitions 1997–2010. In 1997, 43 % of them were “Self-sufficient”, 55 % remained “Jobless”, while 2 % had moved into “Disabled”. Regardless of year of transitions, a high proportion stayed within their previous state. The lowest and the highest stable proportions for “Self-sufficient” were 77 and 90 %, respectively. The corresponding proportions for “Jobless” were 21 and 72 % and for “Disabled” 63 and 76 %.

**Table 3:** Transitions of all jobless in 1995 between three states 1997, 2001, 2005, and 2008, respectively, into following state in 2001, 2005, 2008, and 2010 or censored (Column per cent for each subgroup)

From		1997			2001			2005			2008		
		*n* = 622,170			*n* = 598,766			*n* = 581,377			*n* = 568,797		
		S	J	D	S	J	D	S	J	D	S	J	D
To		43	55	2	65	28	7	64	23	12	70	17	13
2001													
S		**81**	50	14									
J		13	**38**	13									
D		4	7	**69**									
C		2	5	4									
2005													
S		**77**	49	13	**80**	34	13						
J		13	**29**	11	13	**49**	11						
D		7	13	**69**	6	11	**74**						
C		4	9	7	1	7	3						
2008													
S		**80**	53	15	**83**	41	17	**90**	37	14			
J		8	**21**	9	9	**35**	9	6	**50**	8			
D		7	14	**67**	6	13	**69**	3	7	**76**			
C		5	11	9	3	11	5	1	6	2			
2010													
S		**78**	51	15	**81**	40	18	**87**	36	17	**90**	21	11
J		11	**24**	11	11	**36**	13	9	**49**	15	8	**72**	14
D		5	13	**63**	5	11	**63**	2	7	**65**	2	3	**73**
C		6	13	11	3	13	6	1	8	3	>0	4	2

Bolded cells indicate numbers that remained within the same state

*S* self-sufficient (employed, long-term parental leave, and students), *J* jobless (long-term unemployed, unemployed without welfare assistance and social assistance), *D* disabled (long-term sickness absence and disability pension), *C* censored (diseased, emigrated, or old-age pension)

Table 3 also shows transitions between two periods, where the diagonal describes two consecutive periods. The within-state transition for “Self-sufficient” showed a somewhat increasing trend from 81 % in 2001 to 90 % in 2010. For “Disabled”, the within-state transitions varied between 69 % in 2001 to 76 % in 2008. For all three states, the probability of staying within the same state was higher in the last time period (2008–2010) compared to the first period (1997–2001). The most notable change was for “Jobless” where 38 % stayed in the same state in the first period and 72 % in the last period.

### Crude and Adjusted Transition Probabilities

The overall trend was that the largest number of individuals moved from “Jobless” into “Self-sufficient” and some into “Disability”. However, these results may be affected by the distribution of other factors such as socio-economic variables, health status, or labour market conditions. These variables are included in Table 4, where possible heterogeneities were estimated by adding two conditional regressions to the original regression based on previous state. Model 1 used only the previous state to estimate the current state, whereas models 2 and 3 included additional explanatory variables. In all models, “Jobless” was used as the reference group (Table 4).

**Table 4:** Odd Ratios OR and 95 % confidence intervals (CI) for being in one of four states given previous state belonging, among all long-term unemployed in 1995 (*N* = 638,622)

	Self-sufficient (ref: jobless)	Disabled (ref: jobless)	Censored (ref: jobless)
	OR (95 % CI)	OR (95 % CI)	OR (95 % CI)
Model 1			
Intercept	0.82 (0.82–0.82)	0.10 (0.10–0.10)	0.08 (0.08–0.08)
State in previous period (ref: jobless)			
Self-sufficient	10.94 (10.87–11.01)	3.77 (3.72–3.82)	1.46 (1.43–1.49)
Disabled	1.40 (1.37–1.42)	68.66 (67.57–69.78)	2.34 (2.26–2.42)
Model 2			
Intercept	1.20 (1.19–1.21)	0.06 (0.06–0.06)	0.06 (0.06–0.06)
State in previous period (ref: jobless)			
Self-sufficient	9.36 (9.3–9.42)	3.90 (3.85–3.96)	1.42 (1.39–1.44)
Disabled	1.39 (1.36–1.41)	65.31 (64.26–66.38)	2.44 (2.36–2.52)
Sex (ref: male)			
Female	1.17 (1.16–1.17)	1.56 (1.54–1.58)	0.97 (0.95–0.98)
Age (ref: 20–25)			
26–30	0.75 (0.74–0.76)	1.26 (1.24–1.28)	0.89 (0.87–0.91)
31–35	0.65 (0.64–0.65)	1.47 (1.45–1.49)	0.79 (0.77–0.81)
36–40	0.58 (0.58–0.59)	1.73 (1.71–1.76)	0.74 (0.73–0.76)
Education (ref: high school)			
Lower	0.58 (0.57–0.58)	1.09 (1.08–1.10)	1.13 (1.11–1.15)
Higher	1.47 (1.46–1.48)	0.81 (0.80–0.82)	2.04 (2.00–2.08)
Country of birth (ref: born in Sweden)			
Other country than Sweden	0.66 (0.66–0.67)	0.82 (0.81–0.83)	2.01 (1.98–2.05)
Model 3			
Intercept	1.14 (1.14–1.15)	0.02 (0.02–0.02)	0.04 (0.04–0.04)
State in previous period (ref: jobless)			
Self-sufficient	9.88 (9.8–9.96)	2.64 (2.60–2.68)	1.22 (1.19–1.24)
Disabled	1.53 (1.5–1.56)	49.37 (48.45–50.3)	2.51 (2.42–2.60)
Sex (ref: male)			
Female	1.19 (1.18–1.19)	1.56 (1.54–1.58)	0.98 (0.97–1.00)
Age (ref: 20–25)			
26–30	0.75 (0.74–0.75)	1.25 (1.23–1.27)	0.87 (0.86–0.89)
31–35	0.64 (0.64–0.65)	1.45 (1.43–1.48)	0.77 (0.75–0.78)
36–40	0.58 (0.57–0.58)	1.70 (1.67–1.73)	0.72 (0.7–0.73)
Education (ref: high school)			
Lower	0.57 (0.56–0.57)	1.04 (1.03–1.06)	1.1 (1.08–1.120)
Higher	1.48 (1.47–1.49)	0.83 (0.82–0.84)	2.06 (2.02–2.10)
Country of birth (ref: born in Sweden)			
Other country than Sweden	0.66 (0.65–0.66)	0.81 (0.80–0.82)	1.95 (1.92–1.98)
Inpatient care (ref: no inpatient care)			
≤Median	0.86 (0.85–0.87)	1.64 (1.61–1.67)	0.54 (0.52–0.56)
>Median	0.65 (0.64–0.66)	3.13 (3.07–3.18)	1.34 (1.30–1.38)
Period unemployment rate (ref: 1995–1997 increasing unemployment)			
1997–2001 decreasing	1.44 (1.43–1.45)	5.73 (5.60–5.86)	2.8 (2.74–2.86)
2001–2005 increasing	0.82 (0.81–0.83)	5.93 (5.79–6.07)	2.31 (2.26–2.37)
2005–2008 decreasing	1.29 (1.27–1.3)	5.18 (5.06–5.31)	2.23 (2.17–2.29)
2008–2010 increasing	0.86 (0.85–0.87)	2.62 (2.55–2.69)	0.95 (0.92–0.99)

Model 1: *n* = 3,009,732, −2*LL* = 4,548,687, *R* ^2^ = 0.35

Model 2: *n* = 3,009,732, −2*LL* = 4,406,400, *R* ^2^ = 0.38

Model 3: *n* = 3,009,732, −2*LL* = 4,306,862, *R* ^2^ = 0.40

In Model 1, the odds ratio (OR) for being in “Self-sufficient” were almost 11 times higher for those previously in “Self-sufficient”, compared to those previously in the “Jobless” state. The corresponding OR for transition from” Disabled” to “Self-sufficient” was 1.40. The OR for transition from “Self-sufficient” to “Disabled” was, compared to the reference group (“Jobless”), 3.77 and the corresponding OR from the state “Disabled” was 68.66. Thus, the two outcomes, “Self-sufficient” and “Disabled”, showed positive associations (OR > 1) for remaining in the corresponding state (Table 4, Model 1).

The estimated effects of socio-demographic conditions showed, with the exception for sex, different signs for the three outcome states (Table 4, Model 2). Being female and having higher education were positively associated with “Self-sufficient”. For “Self-sufficient”, negative associations (OR < 1) were shown for higher age and being born outside of Sweden. The estimates of being female, higher age, and having lower education were positively associated with transition to “Disabled” (compared to Jobless). Being born outside of Sweden was negatively associated with transition to “Disabled”.

In Model 3, previous inpatient care and dummy variables for changes in unemployment rates during the time periods were introduced (Table 4, Model 3). The estimates for inpatient care showed opposite patterns for the probability of transition into “Self-sufficient” and “Disabled”. For the highest level of inpatient care, the OR for “Self-sufficient” was 0.65, while the corresponding OR for transition into “Disabled” was 3.13.

Further, the years 1997–2001 and 2005–2008 were characterised by decreasing unemployment rates, whereas 2001–2005 and 2008–2010 had increasing unemployment rates (Fig. 2). The estimates for transition to “Self-sufficient” was positive for the two periods following decreasing unemployment (OR_2001_ = 1.44, OR_2008_ = 1.29) but negative for the periods following after increasing unemployment (OR_2005_ = 0.82, OR_2010_ = 0.86). Thus, the probability of transition into “Self-sufficient” co-varied with unemployment levels. This was not the case for “Disabled” where there was a positive association for all time periods. For “Disabled”, the estimates were higher in the earlier time periods (OR_2001_ = 5.73 and OR_2005_ = 5.93) than in the later time periods (OR_2008_ = 5.18 and OR_2010_ = 2.62).

The estimates for previous state showed the same pattern in Model 3 as in Model 1. However, the estimated ORs in Model 3 were somewhat lower compared to in Model 1. The most notable difference between Model 3 and Model 1 were the ORs for staying in “Disabled” (OR = 49.37 vs OR = 68.66, respectively) (Table 4).

### Model Fit

For each model *R* ^2^ was calculated. As expected, the model *R* ^2^ increased with model complexity (*R*_1_^2^ = 0.35, *R*_2_^2^: 0.38 and *R*_3_^2^ = 0.40). Log-likelihood test was used to compare Model 1 versus 2 and Model 2 versus 3. In both tests, the more complex model resulted in better model fit (*p* < 0.05).

### Estimated Transition Probabilities

In order to get a more detailed picture of how previous inpatient care and national unemployment rates were associated with the transitions between the three states, specific transition probabilities were estimated (Table 5). The calculations were stratified by years with two different periods of increasing and decreasing levels of unemployment and the individuals’ previous days with inpatient care. All other variables were fixed at specific values: males, born in Sweden, ages 26–30, with high school education.

**Table 5:** Estimated probabilities for transitions between four states, stratified by national unemployment rate and individuals’ previous days with inpatient care (column per cent for each subgroup)

	Decreasing unemployment rates			Increasing unemployment rates		
	1997–2001			2001–2005		
	No inpatient care	≤Median inpatient care days	>Median inpatient care days	No inpatient care	≤Median inpatient care days	>Median inpatient care days
S → S	89.4	86.7	78.4	82.9	78.8	67.3
S → J	7.3	8.3	9.9	11.9	13.2	15.0
S → D	2.4	4.5	10.2	4.1	7.4	15.9
S → C	0.8	0.5	1.5	1.1	0.7	1.9
J → S	50.3	45.8	34.5	36.8	32.5	23.2
J → J	40.8	43.2	43.2	52.4	53.9	51.0
J → D	5.1	8.8	16.8	6.7	11.4	20.5
J → C	3.8	2.2	5.4	4.0	2.2	5.3
D → S	20.4	12.6	5.6	12.5	7.4	3.2
D → J	10.8	7.8	4.6	11.6	8.0	4.6
D → D	66.3	78.6	88.3	73.7	83.7	91.0
D → C	2.5	1.0	1.4	2.2	0.8	1.2

	Decreasing unemployment rates			Increasing unemployment rates		
	2005–2008			2008–2010		
	No inpatient care	≤Median inpatient care days	>Median inpatient care days	No inpatient care	≤Median inpatient care days	>Median inpatient care days
S → S	88.7	85.9	77.5	85.9	83.1	75.5
S → J	8.2	9.2	11.0	11.8	13.3	16.1
S → D	2.4	4.5	10.2	1.8	3.3	7.6
S → C	0.7	0.4	1.3	0.5	0.3	0.8
J → S	48.1	43.6	33.0	40.3	36.3	28.1
J → J	43.7	46.1	46.2	54.9	57.4	59.0
J → D	4.9	8.5	16.2	3.1	5.4	10.5
J → C	3.3	1.8	4.6	1.7	1.0	2.5
D → S	20.0	12.4	5.5	22.4	14.6	6.8
D → J	11.9	8.6	5.1	20.0	15.1	9.4
D → D	65.9	78.1	88.1	56.0	69.6	82.7
D → C	2.2	0.9	1.3	1.6	0.6	1.0

Estimates for males, born in Sweden, age 26–30, with high school education

*S* self-sufficient (employed, long-term parental leave, and students), *J* jobless (long-term unemployed, unemployed without welfare assistance and social assistance), *D* disabled (long-term sickness absence and disability pension), *C* censored (diseased, emigrated, or old-age pension)

The highest estimated probabilities of young men remaining within the state were seen for “Self-sufficient” (min = 67.3 %, max = 89.4 %) and “Disabled” (min = 56.0 %, max = 91.0 %). The corresponding values for “Jobless” were 40.8 and 59.0 %, respectively (Table 5).

In all time periods and from all states, there was a negative association between the number of days in inpatient care and moving to the state “Self-sufficient”. However, in most cases the estimated probabilities for moving to “Self-sufficient” were lower after a period of increasing unemployment (Table 5). The probabilities of males’ transition from “Self-sufficient” into the state “Jobless” increased with inpatient care (Table 5). This was the case also for women (figures not shown). For the “Jobless” who remained in that state, inpatient care only marginally affected the transition probabilities.

Further, the transitions between “Jobless” into “Disabled” increased for men with previous inpatient care (Table 5). The same was true for women, although with even larger differences between duration of inpatient care (figures not shown). However, both the highest and lowest estimated probabilities for transitions from “Jobless” into “Disabled” among men were found in periods with increasing unemployment (2005 respectively 2010). The same was noted among women (figures not shown). Remaining in the state “Disabled” was positively associated with levels of inpatient care but was less associated with the national level of unemployment (Table 5).

## Discussion

In this study, prospective cohort analyses were conducted of the some 640,000 jobless women and men aged 20–40 in Sweden in 1995. They were followed prospectively for 15 years regarding their labour market position. The focus was on transitions between the three long-term states: “Jobless”, “Self-sufficient”, and “Disabled”.

A majority of the “Jobless” in 1995 were later in the “Self-sufficient” category and only relatively few in the “Disabled” category. The study also showed that the transitions were rather stable over the different time periods for the two states “Self-sufficient” and “Disabled” and less so for the state “Jobless”. The general implication of this is in line with previous analyses of unemployed populations (Andrén 2008; Bäckman and Franzén 2007), showing that large shares of jobless individuals moved in and out of the labour market rather than into long-term sickness absence or disability pension (i.e. the state “Disabled”).

The most notable change was for “Jobless”, where 38 % were in the same state in the first period and 72 % in the last period. There may be several possible reasons for the change in transition frequencies among the “Jobless”. It may have to do with age selection as those who enter the state “Jobless” in later periods have reached a higher age which may reduce their possibility to move into another state (Mastekaasa 1996). It may also be an effect of the cumulative unemployment duration among those who remained in the Jobless state (Alba-Ramírez et al. 2007). Further, the incentives for return to work may in some cases be limited, depending on the marginal income difference to being on social security compensation compared to a low-income job (Allebeck snd Mastekaasa 2004; Hall and Hartman 2010; Henrekson and Persson 2004; Hesselius 2007; Larsson 2006).

The fact that “Disabled” was a stable state is not surprising since disability pension is often permanent and long-term sickness absence is a risk factor for disability pension (Helgesson et al. 2012). Neither is it surprising that “Self-sufficient” was generally a stable state as the alternative labour market positions are generally less common. This has also been confirmed by studies on mobility between work and unemployment (Alba-Ramírez et al. 2007; Steiner 2001).

However, the transition probabilities were also significantly affected by the socio-economic factors sex, age group, education, and country of birth. This indicates that socio-economic information is relevant for predicting individual’s future labour market positioning. Nevertheless, the previous state was the best predictor of subsequent labour market position. This is in line with other multi-state analyses that have shown that individual factors affect the transitions (Øyeflaten et al. 2012, 2014; Pedersen et al. 2012, 2014). Particularly, sex, age, educational level, and country of birth have been shown to be of importance but with varying effects for different study populations and for different outcomes (Øyeflaten et al. 2012, 2014; Pedersen et al. 2012, 2014). However, as in the case of the present study, these studies also found that the previous state was the best predictor for current outcome.

The factors inpatient care and national unemployment rates added further improvement to the used model. “Jobless” individuals who had experienced inpatient care before the follow-up period had a much higher probability of transition into “Disabled” and also a lower transition probability into “Self-sufficient”. On the other hand, individuals that remained in the state “Jobless” were less affected by previous health status than those who transited to the two other states. The result may indicate that individuals who are healthy among those out of work return to work, while those who have previous health problems move into long-term sickness absence or disability pension. To the best of our knowledge, this has not been shown in previous studies with multiple outcomes. However, these results are in line with a study on differential health-related exclusion from the labour market during an economic recession (Burström et al. 2012).

Several recent publications have adopted multi-state models to describe transitions between health-related states and subsequent labour market positioning (Lie et al. 2008; Øyeflaten et al. 2012, 2014; Pedersen et al. 2012, 2014). In most of these studies, the authors differentiated between individuals’ health status by controlling for the sick leave diagnosis (Øyeflaten et al. 2012, 2014; Pedersen et al. 2012, 2014). In the present study, individuals’ previous health status instead was assessed by information on inpatient care. In line with previous results, we found that ill health was negatively associated with probabilities of returning to work and increased the probabilities of sickness absence or disability pension (Øyeflaten et al. 2014; Pedersen et al. 2012, 2014).

The present study found that during periods of reduced unemployment rates, the transition rates into “Self-sufficient” were higher compared to periods of increased unemployment rates. The role of unemployment for transition into “Disabled” did not follow a distinct pattern in this cohort. One interpretation of the distinct effect of previous health is related to a continuous health selection into being unemployed or not in gainful employment. Some individuals may suffer from previous conditions of ill health and these may be reinforced by having a marginalised labour market position. This kind of selection has been shown in previous research (Browning et al. 2003; Helgesson et al. 2012; Janlert and Hammarström 2009; Kokko et al. 2000; Milner et al. 2014; Montgomery et al. 1996; Schmitz 2011). On the other hand, macro-level factors such as unemployment rate have been shown to affect labour market transitions in a few studies (Askildsen et al. 2005; Burström et al. 2012; Holland et al. 2011a; Holland et al. 2011; Lidwall et al. 2004).

In 2003, the Swedish government presented a declaration to reduce sickness absence rates by 50 % until 2008. This leads to stricter practices regarding access to sickness insurance. Also, other changes were introduced, such as national diagnosis-specific guidelines for duration of sickness absence in 2007 and in 2008 that absentees’ work capacity was to be assessed at the latest at day 90, 180, and 364 by the Social Insurance agency and introduction of maximum time of sick leave days (usually one year during an 18-month period) (Lidwall and Marklund 2011; Skånér et al. 2011). These changes were followed by a decline in the sickness absence rates between 2003 and 2010. Overall, these changes were implemented to decrease the inflow and increase the outflow into from the sickness insurance system, thus aiming to increase self-sufficiency. The fact that the present study showed higher outflow from the state “Disabled” to “Self-sufficiency” and that the flow from “Jobless” to “Disabled” was relatively stable indicates differential effects of policy changes on individuals with and without reduced health. However, macro-economic conditions were also shown to influence the flows in and out of the state “Disabled”. Although the costs for labour market policies as well as the distribution between different activities have varied over time, the general access of individuals to employment activities and unemployment insurances have not changed throughout the studied period (Forslund et al. 2011).

## Methodological Considerations

Using data from high-quality nationwide registers (Ludvigsson et al. 2011; SCB 2011), each individual could be categorised into states and followed up annually. Compared to studies based on samples, this is an advantage since there is no loss of information due to dropouts. Another strength is that the used registers contain more reliable information on incomes and hospital care than self-reported data. Further, transitions in different socio-economic groups, health conditions and time periods could be analysed due to the large cohort.

However, categorising individuals into mutually exclusive groups introduced risks of miss classification. The present study classified individuals into two steps. This could potentially result in heterogeneity where individuals within the same state differed in terms of proximity to the labour market. For example, individuals on parental leave and students are different from the much larger group of employed in the state called “Self-sufficient”, particularly in sex and age distributions. Treating these as separate groups would have led to six rather than four states. However, the used amalgamation was motivated by the need to construct a manageable number of possible transitions.

Further, we used annual information of the individual’s sources of incomes to categorise the different states, as that was the nature of data we had access to. Therefore, an individual categorised into one specific state (by main sources of income) could potentially also have some income from other states in the same year. This could be a source of misclassification of the transitions between different states. However, since we aimed to investigate long-term conditions (i.e. groups with distinct types of incomes), we argue that using annual level data is relevant for this aim.

Related, our definition of time was on annual level. We did not, however, include all available years in the empirical analyses; instead, we analysed changes according to documented unemployment cycles. Therefore, the results should be interpreted as empirical transition probabilities between states at specific time points, rather than transition intensities at any points in time. Further details about this distinction are discussed by Andersen and Perme (Andersen and Perme 2008).

Information on inflow into specific employment programmes at different points in time was not available. As different labour market programmes may have different effects on the transition rates (Sianesi 2008), possible interpretations of intervention effects are limited. Regarding previous health problems, we only had access to information about inpatient care for all of the studied years; this can be regarded as both a limitation and a strength. This means that we mainly captured the more serious morbidity panorama, while most medical disorders are treated in outpatient care.

Most studies of transitions between different labour market states, including the present one, were conducted in Scandinavian countries, having compulsory social security systems. National differences in social security systems may reduce the degree to which our results are applicable to other contexts.

Modelling transitions as a first-order Markov model provided a theoretical framework and a practical instrument for handling the complexity of the data, including a multitude of labour market positions and the longitudinal aspect. However, deviations from the Markov model were made as the used time intervals during follow-up were not equally spaced and additional explanatory variables were added. The unequal length of time between points of measurement was motivated by the assumption that national unemployment levels were of importance for the outcome.

Further research should be conducted about how transition rates of different groups are affected by specific social policy changes and on different paths into long-term unemployment.

## Conclusions

Among the “Jobless” in 1995, transitions into “Self-sufficient” increased over time. Higher level of national unemployment decreased the transition probability from “Jobless” into “Self-sufficient”. Staying in the state “Jobless” was more likely among individuals with previous ill health than among others and this was more pronounced in periods with high national unemployment. However, the best predictor for transition into subsequent state was the previous state. For this reason, it can be concluded that the transition rates were relatively stable over time. The results of the study support both theories of health selection and of general labour market effect on future state among long-term unemployed individuals.

The study indicated that further attention should be given to long-term unemployed individuals with a history of health-related problems when considering labour market programmes and interventions.
